# Behavioral characterization of mice overexpressing human dysbindin-1

**DOI:** 10.1186/s13041-014-0074-x

**Published:** 2014-10-09

**Authors:** Norihito Shintani, Yusuke Onaka, Ryota Hashimoto, Hironori Takamura, Tsuyoshi Nagata, Satomi Umeda-Yano, Akihiro Mouri, Takayoshi Mamiya, Ryota Haba, Shinsuke Matsuzaki, Taiichi Katayama, Hidenaga Yamamori, Takanobu Nakazawa, Kazuki Nagayasu, Yukio Ago, Yuki Yagasaki, Toshitaka Nabeshima, Masatoshi Takeda, Hitoshi Hashimoto

**Affiliations:** Laboratory of Molecular Neuropharmacology, Graduate School of Pharmaceutical Sciences, Osaka University, 1-6 Yamadaoka, Suita, Osaka, 565-0871 Japan; Molecular Research Center for Children’s Mental Development, United Graduate School of Child Development, Osaka University, Kanazawa University, Hamamatsu University School of Medicine, Chiba University and University of Fukui, 2-2 Yamadaoka, Suita, Osaka, 565-0871 Japan; Department of Psychiatry, Graduate School of Medicine, Osaka University, 2-2 Yamadaoka, Suita, Osaka, 565-0871 Japan; Department of Molecular Neuropsychiatry, Graduate School of Medicine, Osaka University, 2-2 Yamadaoka, Suita, Osaka, 565-0871 Japan; Department of Chemical Pharmacology, Graduate School of Pharmaceutical Sciences, Meijo University, 150 Yagotoyama, Tempaku-ku, Nagoya, 468-8503 Japan; Molecular Brain Science, United Graduate School of Child Development, Osaka University, Kanazawa University, Hamamatsu University School of Medicine, Chiba University and University of Fukui, 2-2 Yamadaoka, Suita, Osaka, 565-0871 Japan; Anatomy and Neuroscience, Graduate School of Medicine, Osaka University, 2-2 Yamadaoka, Suita, Osaka, 565-0871 Japan; iPS Cell-based Research Project on Brain Neuropharmacology and Toxicology, Graduate School of Pharmaceutical Sciences, Osaka University, 1-6 Yamadaoka, Suita, Osaka, 565-0871 Japan; Laboratory of Medicinal Pharmacology, Graduate School of Pharmaceutical Sciences, Osaka University, 1-6 Yamadaoka, Suita, Osaka, 565-0871 Japan; Department of Mental Disorder Research, National Institute of Neuroscience, National Center of Neurology and Psychiatry, 4-1-1 Ogawahigashicho, Kodaira, Tokyo, 187-8502 Japan; Department of Regional Pharmaceutical Care & Sciences, Graduate School of Pharmaceutical Sciences, Meijo University, 150 Yagotoyama, Tenpaku-ku Nagoya, 468-8503 Japan

**Keywords:** Dysbindin, *DTNBP1*, Dystrobrevin binding protein 1, Psychiatric disorder, Schizophrenia, Transgenic mice, Behavior, Methamphetamine, Phencyclidine, Immediate-early gene

## Abstract

**Background:**

The dysbindin-1 gene (*DTNBP1*: dystrobrevin binding protein 1) is a promising schizophrenia susceptibility gene, known to localize almost exclusively to neurons in the brain, and participates in the regulation of neurotransmitter release, membrane-surface receptor expression, and synaptic plasticity. Sandy mice, with spontaneous *Dtnbp1* deletion, display behavioral abnormalities relevant to symptoms of schizophrenia. However, it remains unknown if dysbindin-1 gain-of-function is beneficial or detrimental.

**Results:**

To answer this question and gain further insight into the pathophysiology and therapeutic potential of dysbindin-1, we developed transgenic mice expressing human *DTNBP1* (Dys1A-Tg) and analyzed their behavioral phenotypes. Dys1A-Tg mice were born viable in the expected Mendelian ratios, apparently normal and fertile. Primary screening of behavior and function showed a marginal change in limb grasping in Dys1A-Tg mice. In addition, Dys1A-Tg mice exhibited increased hyperlocomotion after methamphetamine injection. Transcriptomic analysis identified several up- and down-regulated genes, including the immediate-early genes *Arc* and *Egr2*, in the prefrontal cortex of Dys1A-Tg mice.

**Conclusions:**

The present findings in Dys1A-Tg mice support the role of dysbindin-1 in psychiatric disorders. The fact that either overexpression (Dys1A-Tg) or underexpression (Sandy) of dysbindin-1 leads to behavioral alterations in mice highlights the functional importance of dysbindin-1 *in vivo*.

## Background

Dysbindin-1 (dystrobrevin binding protein 1) is an evolutionary conserved 40-kDa coiled-coil-containing protein that binds to dystrobrevin and localizes exclusively to neurons in the brain [1]. Dysbindin-1 has been shown to participate in biogenesis of lysosome-related organelles complex 1, which regulates trafficking to lysosome-related organelles [2], regulation of neurotransmitter release [3-5], membrane surface expression of glutamate NMDA [6] and dopamine D2 [7,8] receptors, and synaptic plasticity [6,9].

Genetic variations in the human dysbindin-1 gene (*DTNBP1*) have been shown to be associated with schizophrenia [5,10], bipolar disorder [11], and methamphetamine (METH) psychosis [12], as well as neurocognitive functions in healthy subjects [13,14]. In postmortem brain from schizophrenic patients, decreased dysbindin-1 expression has been demonstrated in the prefrontal cortex [15], cerebral cortex [16], and intrinsic glutamatergic terminals of the hippocampal formation [17].

Sandy mice completely lack dysbindin-1 protein because of spontaneous deletion of introns 5–7 of the *Dtnbp1* gene in DBA/2 J mice [2]. These mice display a variety of behavioral abnormalities relevant to symptoms of schizophrenia, including hypoactivity, heightened anxiety-like responses, reduced social interaction [18], deficits in both long-term [19] and working memory [20], and impairments in contextual fear conditioning [9]. As potential mechanisms for these behavioral abnormalities, Sandy mice have been shown to exhibit reduced dopamine transmission in the forebrain [18] and destabilization of snapin, which binds to SNAP25 and regulates calcium-dependent exocytosis [19].

The sandy mutation was backcrossed onto a C57BL/6 J background for at least 11 generations to obtain sdy/B6 mice [21]. These mice show schizophrenia-like behaviors including hyperactivity, spatial learning and memory deficits, impaired working memory under challenging conditions, and disruption of dopamine/D2-related mechanisms that regulate cortical function and neuronal excitability [21,22]. sdy/B6 mice also exhibit increased impulsive and compulsive behaviors relevant to psychiatric disorders [23].

Thus, a growing body of evidence implicates dysbindin-1 in psychiatric disorders. However, because of failure to replicate genetic association studies [24], a lack of causal variants with a notable impact on disease risk that might contribute to schizophrenia [25], and methodological difficulties in postmortem brain research due to heterogeneity of tissues with respect to biochemical parameters, lifetime history of medications and physiological status at the time of death [26], it remains unclear how dysbindin-1 functions as a susceptibility gene for these disorders.

A recent study in mice and humans demonstrated an epistatic interaction between catechol-O-methyl transferase (COMT) and dysbindin-1 that modulates prefrontal function, specifically, subjects with reduced function of either COMT or dysbindin-1 show superb physiological performance, whereas those with reductions in both proteins have performance deficits [27].

As dysbindin-1 has both beneficial and detrimental effects in prefrontal cortical function, we performed a gain-of-function study of dysbindin-1 by developing transgenic mice that express the human dysbindin-1 gene (Dys1A-Tg) and we analyzed their behavioral phenotypes.

## Results

### Generation of Dys1A-Tg mice

**Figure 1:**
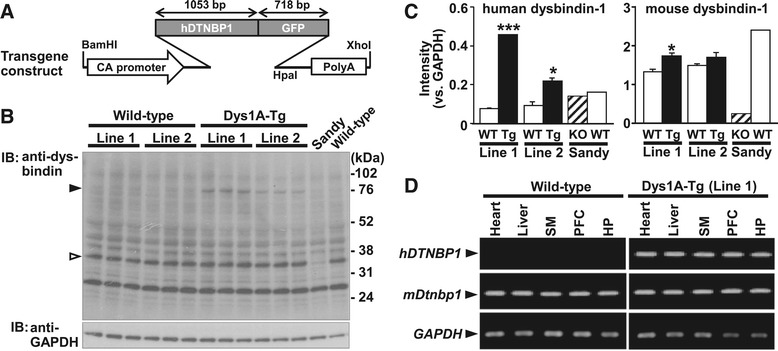
**Generation of Dys1A-Tg mice. (A)** Schematic of the transgene construct (hDTNBP1-GFP) with CA promoter, human dysbindin-1 (*hDTNBP1*) cDNA C-terminally fused to GFP, and Simian virus 40 polyadenylation signal sequence (PolyA). **(B)** Western blot analysis of transgenically expressed human dysbindin-1 and endogenous (mouse) dysbindin-1 protein in Dys1A-Tg mice. Protein lysates prepared from whole brain of adult male Dys1A-Tg mice (lines 1 and 2) and wild-type littermates, a Sandy mouse lacking dysbindin-1 protein, and a wild-type control mouse were subjected to western blot analysis with anti-dysbindin and anti-GAPDH antibodies. Closed and open arrowheads indicate transgene products and endogenous dysbindin-1 protein, respectively. **(C)** The intensity of each band in the western blot **(B)** was quantitated and normalized vs. GAPDH. Data are expressed as mean ± SEM. WT, wild-type; Tg, Dys1A-Tg; KO, knockout. *P < 0.05, ***P < 0.001 vs. wild-type of the same line. **(D)** Semi-quantitative RT-PCR analysis of transgenic human dysbindin-1 and endogenous mouse dysbindin-1 mRNA expression in various tissues of Dys1A-Tg mice and wild-type littermates. *GAPDH* serves as an internal control. *hDTNBP1*, human dysbindin-1; *mDtnbp1*, mouse dysbindin-1; SM, smooth muscle; PFC, prefrontal cortex; HP, hippocampus.

Dysbindin-1 exists as multiple isoforms produced by alternative mRNA splicing. Of these isoforms, dysbindin-1A, -1B, and -1C are commonly expressed transcripts, although dysbindin-1B is not expressed in mice [17]. Here, we constructed a transgene expressing human dysbindin-1A isoform cDNA, C-terminally fused to GFP (hDTNBP1-GFP) [28] under control of the CA promoter, consisting of a modified promoter of the chicken gene for β-actin with a cytomegalovirus immediate-early enhancer [29] (Figure 1A). Pronuclear injection of the transgene into fertilized C57BL/6 J mouse eggs was performed, generating Dys1A-Tg mice expressing human dysbindin-1 protein (Figure 1B). There was no apparent compensatory decrease in endogenous (mouse) dysbindin-1 expression in Dys1A-Tg mice (Figure 1C). This indicated that total expression levels of dysbindin-1 are significantly increased in Dys1A-Tg mice. Since it was revealed that line 1 Dys1A-Tg mice express higher levels of human dysbindin-1, we performed the following experiments in this line. Through subsequent matings with wild-type mice, offspring were obtained at a frequency not significantly different from the expected Mendelian ratio (Dys1A-Tg, 47.4%; *n* = 190; *P* = 0.157 by *χ*^2^ analysis). Dys1A-Tg mice were fertile, and germline transmission of the transgene was confirmed for at least 10 generations, with an apparently normal coat color (data not shown). Reverse transcription-polymerase chain reaction (RT-PCR) analysis detected *hDTNBP1*-*GFP* transcript expression in various tissues of Dys1A-Tg mice, but not in wild-type mice (Figure 1D). Quantitative real-time RT-PCR revealed that *hDTNBP1*-*GFP* mRNA is expressed in the brain cortex in Dys1A-Tg (line 1) mice but not in wild-type mice, whereas mouse *Dtnbp1* mRNA is similarly expressed in the brain cortex in Dys1A-Tg and wild-type mice (*hDTNBP1*-*GFP* mRNA, Dys1A-Tg, 3.77 ± 0.66, n = 5; wild-type, 0.00 ± 0.00, n = 4, *P* = 0.001; mouse *Dtnbp1* mRNA, Dys1A-Tg, 0.91 ± 0.06, n = 5; wild-type, 1.00 ± 0.09, n = 4, *P* = 0.42; normalized to glyceraldehyde-3-phosphate dehydrogenase (GAPDH) mRNA and relative to mouse *Dtnbp1* mRNA in wild-type mice).

### Behavioral characterization of Dys1A-Tg mice under basal conditions

**Figure 2:**
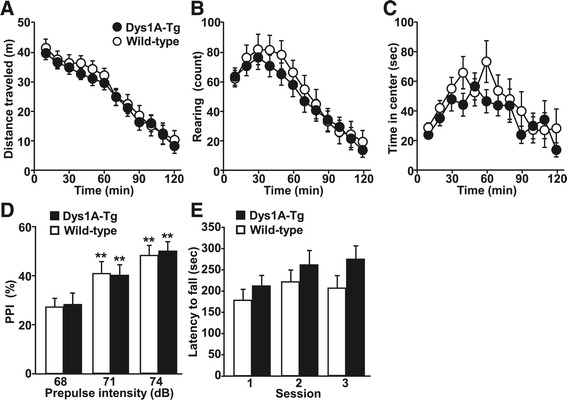
**Essentially normal behavior in Dys1A-Tg mice under basal conditions.** Distance traveled **(A)**, vertical rearing activity **(B)**, and time spent in the center area **(C)** of the open-field test, PPI of the acoustic startle response **(D)**, and latency to fall in the accelerated rotarod test **(E)** were analyzed in Dys1A-Tg (closed symbols and bars) and wild-type (open symbols and bars) mice. Data are expressed as mean ± SEM. Number of mice for each genotype, 19–21 **(A–C)**, 17–19 **(D)**, and 17 **(E)**. Statistical analysis was performed by repeated two-way ANOVA. **P < 0.01 vs. PPI value at 68 db of pre-pulse intensity. There was no significant main effect of genotype in any experiment.

To determine if dysbindin-1 overexpression affects physical and behavioral profiles in mice, we used the standardized SHIRPA (SmithKline Beecham, Harwell, Imperial College, Royal London Hospital, phenotype assessment) screening program [30], which was slightly modified as described [31]. Of the 28 components in the primary SHIRPA screening, a significant difference between Dys1A-Tg and wild-type littermates was observed only in limb grasping behavior (*P* = 0.045, Mann–Whitney *U* test; Table 1). Moreover, *χ*^2^ analysis revealed a significant genotype difference in the number of mice that showed limb grasping behavior (Dys1A-Tg, 72%, n = 18; wild-type, 38%, n = 16; *χ*^2^ = 10.7, P = 0.0011). There were no appreciable differences in the other motor functions, appearance, sensory functions, or anxiety levels between Dys1A-Tg and wild-type mice (Table 1). Further behavioral analyses in secondary screening showed no genotype differences in distance traveled, rearing, and time spent in the center region of the open-field test (Figure 2A − C), pre-pulse inhibition (PPI) levels (Figure 2D), startle responses to main pulses of 120 dB (Dys1A-Tg, 407 ± 53; wild-type: 395 ± 66; arbitrary unit), and latency to fall over in three sessions of the accelerated rotarod test (Figure 2E). These results suggest that under basal conditions, Dys1A-Tg mice have no gross behavioral abnormalities except in limb grasping behavior.

**Table 1:** **SHIRPA primary screening in Dys1A**-**Tg mice**

**Paradigm and examination**		**Wild-type**	**Dys1A-Tg**	**P value**
**Appearance**				
	Body weight (g)	23.4 ± 0.43	23.1 ± 0.40	0.88
	Body position	4.00 ± 0 00	4.00 ± 0.00	N.D.
	Respiration rate	2.00 ± 0.00	2.06 ± 0.06	0.35
	Heart rate	1.06 ± 0.06	1.11 ± 0.08	0.62
	Tremor	0.13 ± 0.09	0.11 ± 0.08	0.90
	Palpebral closure	0.00 ± 0.00	0.00 ± 0.00	N.D.
	Piloerection	0.00 ± 0.00	0.00 ± 0.00	N.D.
	Lacrimation	0.00 ± 0.00	0.00 ± 0.00	N.D.
**Motor function**				
	Spontaneous activity	3.00 ± 0.00	3.00 ± 0.00	N.D.
	Transfer arousal	3.94 ± 0.17	4.00 ± 0.16	0.79
	Gait	0.00 ± 0.00	0.03 ± 0.03	0.35
	Pelvic elevation	2.06 ± 0.06	2.06 ± 0.06	0.93
	Tail elevation	1.81 ± 0.10	1.83 ± 0.09	0.88
	Trunk curl	0.38 ± 0.13	0.44 ± 0.12	0.69
	Limb grasping	0.38 ± 0.13	0.72 ± 0.11	**0.045**
	Grip strength	2.56 ± 0.13	2.72 ± 0.14	0.44
	Righting reflex	0.00 ± 0.00	0.00 ± 0.00	N.D.
	Contact reflex	1.00 ± 0.00	0.97 ± 0.03	0.35
	Negative geotaxis	0.00 ± 0.00	0.00 ± 0.00	N.D.
**Sensory function**				
	Touch escape	1.81 ± 0.10	1.89 ± 0.11	0.64
	Positional passivity	0.03 ± 0.03	0.06 ± 0.06	0.97
	Visual placing	1.88 ± 0.09	1.94 ± 0.06	0.48
	Corneal reflex	1.00 ± 0.00	1.00 ± 0.00	N.D.
	Toe pinch	2.75 ± 0.11	2.72 ± 0.11	0.86
**Anxiety level**				
	Urination	0.38 ± 0.13	0.17 ± 0.09	0.18
	Defecation	2.56 ± 0.49	3.00 ± 0.58	0.75
	Vocalization	0.88 ± 0.09	0.89 ± 0.08	0.90
	Provoked biting	0.88 ± 0.09	0.83 ± 0.09	0.74

Data are expressed as mean ± SEM (Dys1A-Tg, *n* = 18; wild-type, *n* = 16). *P* values were calculated using the Mann–Whitney *U* test. Bold type indicates *P* < 0.05. N.D., not different.

### Behavioral response to acute treatment with METH and phencyclidine in Dys1A-Tg mice

**Figure 3:**
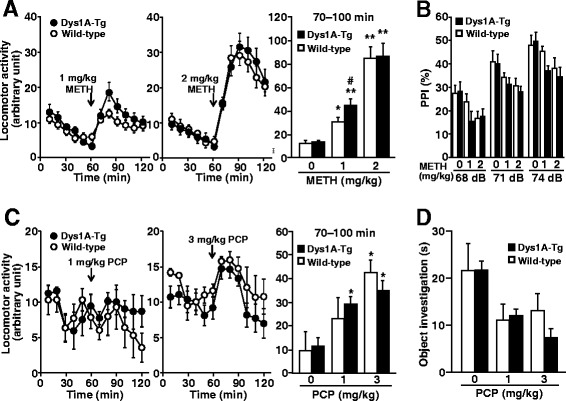
**Slightly increased responses to METH and PCP in Dys1A-Tg mice.** Acute behavioral responses to METH **(A and B)** or PCP **(C and D)** were examined using locomotor **(A and C)**, PPI **(B)**, and object investigation **(D)** tests in Dys1A-Tg (closed symbols and bars) and wild-type (open symbols and bars) mice. METH or PCP were injected at 60 min and cumulative locomotor activity measured for 70–100 min was indicated in bar graphs in **(A)** and **(C)**. Data are expressed as mean ± SEM. Number of mice for each genotype, 14–27 **(A)**, 17–35 **(B)**, 3–8 **(C)**, and 3–5 **(D)**. Statistical analysis was performed by repeated three or two-way ANOVA followed by the Tukey–Kramer post-hoc test. *P < 0.05, **P < 0.01 vs. vehicle of the same genotype. ^#^ *P* < 0.05 vs. wild-type of the same treatment.

Mice administered with psychostimulants (e.g., METH) and non-competitive N-methyl-D-aspartate receptor antagonists (e.g., phencyclidine, PCP) serve as animal models for psychiatric disorders including schizophrenia [32,33]. *DTNBP1* has been implicated as a risk factor for psychiatric disorders such as schizophrenia [9-11,13-20], therefore we examined behavioral responses to acute treatment with METH and PCP in Dys1A-Tg mice. In the open-field test, repeated two-way analysis of variance (ANOVA) revealed a significant time and genotype interaction in METH-induced hyperlocomotion at 1 mg/kg (*F*_11, 572_ = 2.28, *P* = 0.010), while METH at 2 mg/kg had no significant effect on the interaction (*F*_11, 374_ = 0.784, *P* = 0.66; Figure 3A). Dys1A-Tg mice administered with 1 mg/kg METH showed significantly increased total locomotor activity for 30 min immediately after METH administration compared with wild-type mice (*P* < 0.05; Figure 3A *right*). After METH administration, PPI was significantly disrupted (treatment effect, *F*_2, 272_ = 6.75, *P* = 0.0016) but no significant genotype effect was identified (*F*_1, 272_ = 1.27, *P* = 0.26; repeated three-way ANOVA; Figure 3B). Two-way ANOVA revealed a significant effect of PCP on locomotor activity (*F*_2,25_ = 16.6, *P* < 0.0001) but not genotype effect (*F*_1, 25_ = 0.005, *P* = 0.94; two-way ANOVA; Figure 3C). The novel object investigation test was subsequently performed using mice that received PCP (Figure 3D). In this test, exploratory behavior towards a novel object is assessed. Two-way ANOVA revealed a significant effect of PCP (*F*_2, 19_ = 7.74, *P* = 0.0035) but not the interaction of PCP and genotype (*F*_2,19_ = 0.718, *P* = 0.50).

### Behavioral response to chronic PCP treatment in Dys1A-Tg mice

**Figure 4:**
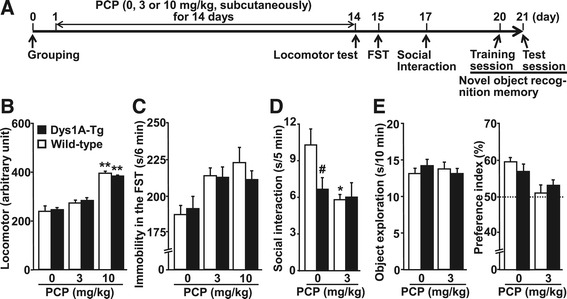
**Effects of chronic PCP administration in Dys1A-Tg mice. (A)** The experimental schedule consisted of four successive behavioral tests (see Methods for details). **(B**–**E)** PCP was administered subcutaneously daily for 14 days, and locomotor activity measured using a digital counter system with an infrared sensor for 90 min **(B)**, immobility time in the FST **(C)**, duration of social interaction between two unfamiliar test mice of the same genotype and treatment **(D)**, and object exploration time and preference index in the test session of the novel object recognition memory test on day 21 **(E)** were determined in Dys1A-Tg (closed bars) and wild-type (open bars) mice. Data are expressed as mean ± SEM. Number of mice for each genotype is 12–14 obtained from three independent cohorts. Statistical analysis was performed by two-way ANOVA followed by the Tukey–Kramer post hoc test. *P < 0.05 vs. vehicle of the same genotype, ^#^ *P* < 0.05 vs. wild-type of the same treatment.

We also examined behavioral responses to chronic treatment with PCP for 14 days in Dys1A-Tg mice. To reduce the number of animals, mice were subjected to a battery of behavioral tests (Figure 4A). Dys1A-Tg and wild-type littermates (*n* = 12–14) were subcutaneously injected with PCP (3 or 10 mg/kg) or vehicle (saline) once a day for 14 days, as previously reported [34]. PCP-induced increases in locomotion were not different between Dys1A-Tg and wild-type mice (Figure 4B). In the FST, two-way ANOVA revealed a significant effect of PCP (*F*_2, 70_ = 12.5, *P* < 0.0001) but not interaction between PCP and genotype (*F*_2, 70_ = 0.848, *P* = 0.43; Figure 4C). As several mice injected with PCP at 10 mg/kg showed abnormal behavior such as increased locomotion in home cages, we subsequently performed the social interaction and novel object recognition tests in mice injected with 3 mg/kg PCP or vehicle only. In wild-type mice, as expected, PCP (3 mg/kg) significantly impaired social interaction and preference index in the novel object recognition test (Figure 4D, E). In contrast, Dys1A-Tg mice showed mildly attenuated responses to chronically administered PCP, but these did not reach statistically significant levels except for the social interaction test. In the social interaction test (Figure 4D), two-way ANOVA revealed no significant effect of PCP (*F*_1, 48_ = 1.28, *P* = 0.26) but identified a significant interaction between PCP and genotype (*F*_1, 48_ = 4.24, *P* = 0.045). The post-hoc Tukey–Kramer test showed that chronic PCP reduces social interaction in wild-type mice only (*P* < 0.05), and there is a significant difference between Dys1A-Tg and wild-type mice injected with vehicle (*P* < 0.05). In the novel object recognition test, mice first freely explore two objects (training session), and memory retention for the objects evaluated after 24 h (test session). As statistical analysis showed no significant differences in training session behavior (data not shown), test session results are shown (Figure 4E). Two-way ANOVA revealed a significant PCP effect for the preference index (*F*_1, 48_ = 11.8, *P* = 0.0012) but not interaction between PCP and genotype (*F*_1,48_ = 1.58, P = 0.22).

### Altered gene expression in Dys1A-Tg mouse brain

As dysbindin-1 is involved in transcriptional regulation [28,35], we performed gene expression profiling in the prefrontal cortex, hippocampus, and striatum of Dys1A-Tg mice, using the Affymetrix GeneChip. Significantly different expression between Dys1A-Tg and wild-type mice was detected in 13 genes (Table 2). The expression of other genes including endogenous (mouse) *Dtnbp1* did not statistically significantly differ between the two genotypes. We could not detect the transgenic gene (human) *DTNBP1* because of species difference in nucleotide sequence. TRAF2 and NCK interacting kinase gene (*Tnik*) was significantly increased in both the prefrontal cortex and striatum of Dys1A-Tg mice. Ten out of 13 genes were decreased in Dys1A-Tg mice, with the most prominent being the immediate-early gene activity regulated cytoskeletal-associated protein (*Arc*) and early growth response 2 (*Egr2*) in the prefrontal cortex (0.29- and 0.48-fold vs. wild-type, respectively).

**Table 2:** **Genes with significantly altered expression in the brain of Dys1A**-**Tg mice**

**Brain region/changes in Dys1A-Tg**	**Gene name**	**Gene symbol**	**UniGene ID**	**Fold change vs. wild-type**	**Signal intensity**	
					**Wild-type**	**Dys1A-Tg**
**Prefrontal cortex**						
*Increased*	TRAF2 and NCK interacting kinase	Tnik	Mm.126193	1.3 ± 0.1	3765 ± 169	4742 ± 195
*Decreased*	Kruppel-like factor 10	Klf10	Mm.4292	0.6 ± 0.0	1055 ± 21	621 ± 31
	Activity regulated cytoskeletal-associated protein	Arc	Mm.25405	0.3 ± 0.1	2194 ± 442	644 ± 156
	early growth response 2	Egr2	Mm.290421	0.5 ± 0.1	633 ± 76	307 ± 86
	RIKEN cDNA 5330406 M23 gene	5330406M23Rik	Mm.109877	0.7 ± 0.0	2301 ± 117	1502 ± 86
	Myelin transcription factor 1-like	Myt1l	Mm.253067	0.6 ± 0.0	1926 ± 154	1224 ± 77
	RIKEN cDNA C130075A20 gene	C130075A20Rik	––	0.5 ± 0.0	643 ± 71	348 ± 21
	Integrin beta 1 binding protein 1	Itgb1bp1	Mm.352231	0.5 ± 0.0	343 ± 23	172 ± 14
**Hippocampus**						
*Increased*	G protein-coupled receptor 178	Gpr178	Mm.297552	1.4 ± 0.1	1512 ± 71	2169 ± 76
	Chemokine (C-C motif) ligand 21b	Ccl21b	Mm.220853	1.7 ± 0.1	874 ± 78	1503 ± 65
*Decreased*	zinc finger, MYM domain containing 1	Zmym1	Mm.273806	0.7 ± 0.0	2050 ± 64	1398 ± 75
**Striatum**						
*Increased*	TRAF2 and NCK interacting kinase	Tnik	Mm.126193	1.3 ± 0.1	3418 ± 220	4587 ± 232
*Decreased*	DNA segment, Chr 4, Wayne State University 53, expressed	D4Wsu53e	Mm.331964	0.7 ± 0.1	2878 ± 230	1863 ± 165
	Kruppel-like factor 2 (lung)	Klf2	Mm.26938	0.6 ± 0.1	414 ± 62	244 ± 27

Transcripts that satisfy the microarray quality criterion (quality index > 0.3) were analyzed. Signal intensity data are expressed as mean ± SEM of three pools, each from three mice.

## Discussion

We aimed to gain insight into the role of dysbindin-1 in psychiatric disorders. To this end, we first generated Dys1A-Tg mice expressing human *DTNBP1* and then analyzed their phenotypes. In order to investigate the function of dysbindin-1 relevant to clinical application, human *DTNBP1* was chosen as a transgene. Dys1A-Tg mice were born viable in the expected Mendelian ratios, apparently normal and fertile. Primary screening of behavior and function using the SHIRPA protocol showed a marginal change in limb grasping in Dys1A-Tg mice. They also exhibited increased hyperlocomotion after METH administration. In the brain of Dys1A-Tg mice, transcriptomic analysis identified several up- and down-regulated genes, including the immediate-early genes *Arc* and *Egr2*.

Among two lines of Dys1A-Tg mice generated, total levels of dysbindin-1 (human and mouse dysbindin-1) were considered to be significantly higher in line 1 while those in line 2 were only slightly higher than wild-type mice (Figure 1C). Therefore, we performed the following behavioral and gene expression experiments in line 1. However, we could not rule out the possibility that the obtained results in the present study might be attributed to the disruption of other genes where the transgene was inserted.

In the social interaction test, which was conducted on day 17 in our test battery, we observed impairments in Dys1A-Tg mice that received vehicle (saline) for 14 days compared with wild-type mice (Figure 4D). Although PCP significantly decreased social interaction in wild-type mice, it did not further decrease the behavior in Dys1A-Tg mice. Interpretation of these results is difficult but may be related to detrimental effect in Dys1A-Tg mice caused by repeated vehicle administration for 14 days. As our previous study showed that the FST lasts for only 3 days after the last PCP injection in C57BL6/J mice [34], and it is necessary to minimize test interactions [36,37], we designed the present behavioral test battery. However, since we did not use a washout period following chronic PCP treatment, the possibility for residual acute effects of PCP may not be excluded especially in the locomotor test and the FST conducted on day 14 and day 15, respectively, and in the mice treated with PCP at 10 mg/kg.

As discussed above, there is a growing body of evidence implicating dysbindin-1 in psychiatric disorders [9-11,13-20], nevertheless it remains unclear how dysbindin-1 increases susceptibility to these disorders [24-26]. Dysbindin-1-deficient mutant Sandy mice (spontaneous mutant in a DBA/2 J mouse strain) display a variety of behavioral abnormalities relevant to symptoms of schizophrenia [9,18-20], as well as reduced dopamine transmission in the forebrain [18]. sdy/B6 mice (Sandy mutant mice on a C57BL/6 J background) show schizophrenia-like behaviors including hyperactivity, learning and memory deficits, and disruption of dopamine/D2-related mechanisms that regulate cortical function and neuronal excitability [21,22]. These mice also exhibit increased impulsive and compulsive behaviors relevant to psychiatric disorders [23]. The present observations that Dys1A-Tg mice are essentially normal under basal conditions (except for increased limb grasping behavior) and exhibit altered behavioral responses to METH, indicate that dysbindin-1 overexpression does not cause strong detrimental effects under basal conditions but may induce vulnerability toward psychotomimetics. The fact that either overexpression (Dys1A-Tg) or underexpression (Sandy both on DBA/2 J and C57BL/6 J backgrounds) of dysbindin-1 leads to behavioral alterations in mice highlights the functional importance of this protein and the molecular networks in which dysbindin-1 is involved.

Dysbindin-1 is expressed ubiquitously in the body and brain [1], and has been postulated to be implicated not only in psychiatric disorders such as schizophrenia, bipolar disorder, and METH psychosis [5,10-12,15-17], but also peripheral diseases such as type 7 Hermansky-Pudlak syndrome, which is accompanied with oculocutaneous albinism, prolonged bleeding, and pulmonary fibrosis due to abnormal vesicle trafficking to lysosomes and related organelles [2]. Thus, Dys1A-Tg mice may serve as a model for various diseases and complement dysbindin-1-null Sandy mice.

*DTNBP1* variants (including e.g., protective and risk haplotypes) are reported to affect susceptibility to substance-induced psychosis [12], and dysbindin-1 is involved in regulation of synaptic plasticity [6,9], neurotransmitter release [3-5], and membrane surface expression of NMDA and D2 receptors [6-8]. Altogether, it is suggested that dysbindin-1 plays significant roles in neurobehavioral control and psychiatric disorders.

## Conclusions

In summary, we have generated Dys1A-Tg mice expressing human *DTNBP1*. Dys1A-Tg mice are apparently normal and fertile without abnormalities in their coat color, but with a marginal change in limb grasping, slightly exaggerated behavioral response to acutely administered METH. In the brain of Dys1A-Tg mice, expression levels of several genes are altered, including the immediate-early genes, *Arc* and *Egr2*. Our results in Dys1A-Tg mice further suggest a critical role for dysbindin-1 in psychiatric disorders.

## Methods

### Ethics statement

All animal care and handling procedures were performed according to the Guidelines for the Care and Use of Laboratory Animals approved by the Japanese Pharmacological Society, and were approved by the Animal Care and Use Committee of the Graduate School of Pharmaceutical Sciences, Osaka University. All efforts were made to minimize the number of animals used.

### Generation of Dys1A-Tg mice expressing human dysbindin-1

hDTNBP1-GFP consists of *Homo sapiens* dysbindin-1 isoform A (accession no. NP_115498, 351 amino acids), green fluorescent protein (GFP), and a termination codon. A 1,773-bp fragment encoding 591 amino acids of hDTNBP1-GFP was inserted into the *Hap*I site of the pCA-pA vector containing the CA promoter [29], and the transgene construct confirmed by DNA sequencing. Next, a 3.7-kb fragment including hDTNBP1-GFP cDNA was excised by *Bam*HI-*Xho*I digestion, and used to generate Dys1A-Tg mice by pronuclear injections into fertilized C57BL/6 mouse eggs.

Genotypes were determined by PCR using genomic DNA extracted from tail biopsies in extraction buffer (5 mM EDTA, 100 mM Tris–HCl, pH 8.5, 200 mM NaCl, 0.2% SDS, and 200 μg/mL proteinase K). Genotyping was performed on genomic DNA (40 ng) using AmpliTaqGold DNA Polymerase (Applied Biosystems, Foster City, CA, USA) and the following primers located in different exons of the human *DTNBP1* gene (5′-GAC TAA GAA TCC ATG ACA GCA AAT C-3′ and 5′-TTA ATT CTG AGG GAT TTG GAA CCT-3′; product size, 547 bp). The PCR reaction consisted of 40 cycles of denaturation at 94°C for 30 s, annealing at 55°C for 30 s, and elongation at 72°C for 1 min.

Dys1A-Tg mice were backcrossed with female C57BL/6 J mice (Charles River, Osaka, Japan) for at least 10 generations. Wild-type female mice were mated with male Dys1A-Tg mice and 8–20-week-old male offspring were used for experiments. Mice were group housed under a 12-h light–dark cycle (lights on at 8:30 a.m.) with free access to food and water.

### RT-PCR and western blot analyses

RT-PCR was performed as described previously, but with some modifications [38]. Briefly, total RNA was reverse transcribed and cDNA from three mice mixed and subjected to semi-quantitative RT-PCR analysis using Gotaq Hot Start Green Master Mix (Promega, Tokyo, Japan) and the following primers corresponding to different exons of the mouse *Dtnbp1* gene (5′-GAA CCA TTT GCT GCA CCT GGA C-3′ and 5′-GGC CTT CTG TGT GTG CTC TGT ATC G-3′; product size, 157 bp), the human *DTNBP1* gene (5′-GCA GCT CCC AGC TTT AAT CGC AG-3′ and 5′-TGG GCG TGC TCT GCA TCT AGT-3′; product size, 232 bp), and the mouse GAPDH gene, which served as an internal control (5′-GTG TTC CCT ACC CCC AAT GTG-3′ and 5′-TAC CAG GAA ATG AGC TTG AC-3′; product size, 241 bp). To confirm validity of genomic DNA amplification, the mouse *Dtnbp1* gene was amplified using intron 6-specific primers (5′-GCA CTC AGG AGA CCA TGA CA-3′ and 5′-GGT TGA CAC TCT TGC GGA AT-3′; product size, 305 bp). Quantitative real-time RT-PCR was also performed in the same way as mentioned above.

Western blot analysis was performed as described [39,40] using mouse monoclonal anti-dysbindin antibody, which was produced in our laboratory against glutatione S-transferase-fused human dysbindin-1 [28]. Briefly, 20 μg of protein from precipitated brain homogenates were separated on SDS-PAGE and electrotransferred onto Immobilon-P Transfer Membranes (Millipore, Billerica, MA, USA), and then probed with primary antibodies: mouse monoclonal anti-dysbindin antibody (1:1,000; Cell Signaling Technology, Danvers, MA, USA) and mouse anti-GAPDH antibody (1:10,000; Millipore), followed by anti-mouse horseradish peroxidase-conjugated antibody (1:2,000; GE Healthcare, Piscataway, NJ, USA). The intensity of the bands was quantitated with Image J software (National Institutes of Health, MD, USA).

### Drugs and experimental design for behavioral analyses

Drug solutions were administered to mice in a volume of 0.1 mL/10 g body weight. METH and PCP dissolved in saline were acutely injected intraperitoneally or subcutaneously, respectively. Each behavioral study was performed using separate cohorts of mice, except for the novel object investigation test.

In the chronic PCP administration model [34], 6-week-old mice were chronically administered with PCP for 14 consecutive days, and then subjected to a battery of four different behavioral tests: locomotor analysis on day 14 (30 min after the last PCP dose), FST on day 15, social interaction test on day 17, and novel object recognition test on days 20 (training session) and 21 (retention test session). The sequence of this behavioral test battery was fundamentally designed to minimize test interactions, by arranging the least stressful tasks first and more stressful tasks last [36,37], with the exception that the FST was performed the day after the last PCP injection as it has been shown that chronic PCP-increased immobility in the FST lasts for only 3 days in C57BL/6 J mice [34].

### Initial behavioral screening

Fundamental sensory and physical functions of mice were evaluated using SHIRPA [30], with slight modifications as described previously [31].

### Locomotor analysis

The open-field test was performed using the infrared Actimeter system (Panlab, Barcelona, Spain), and distance traveled, vertical rearing activity, and time spent in the center area were measured using Acti-Track software (Panlab), as described previously [41,42]. In the PCP study, locomotor activity was measured using a digital counter system with an infrared sensor (Supermex; Muromachi Kikai Co., Tokyo, Japan), as described previously [43].

### PPI of the acoustic startle response

PPI of the acoustic startle response was measured in a startle chamber (SR-LAB; San Diego Instruments, San Diego, CA, USA), essentially as described previously [44]. PPI was calculated as percentage score for each pre-pulse trial type using the following equation: pre-pulse inhibition (%) = [1 − (startle response for pulse with pre-pulse)/(startle response for pulse alone)] × 100.

### Rotarod test

An accelerating rotarod treadmill (Acceler Rota-Rod 7650; Ugo Basile, Varese, Italy) was used to evaluate motor coordination and learning. Mice were first trained repeatedly at a fixed speed (12 rpm) until the mice were able to stay on the rod for at least 300 s. One day after training, performance on the accelerating (12–30 rpm) rotarod was examined for a maximum recording time of 600 s. Tests were performed once for 3 consecutive days.

### Novel object investigation test

Exploratory behavior towards a novel object was evaluated as described [45]. After 15 min habituation under dim light (40 lx) in an observation cage (28 cm length × 20 cm width × 12 cm height), mice were presented with a novel object (a wooden ball; diameter 5 cm), which was placed in the center of the cage. Duration of object exploratory behavior (sniffing or licking the wooden ball) was measured for 5 min from recordings by trained blinded observers. The test was performed just after locomotor analysis in the same mice treated with acute PCP.

### FST

The FST was performed as described previously [46]. Briefly, behavior of mice in a glass cylinder (19 cm diameter × 25 cm height) containing water (25 ± 1°C) to a depth of 13 cm was videotaped for 6 min, and duration of immobility (making only minimal movements to keep floating) was measured by trained blinded observers. After the test, mice were dried thoroughly with a towel and returned to their home cage.

### Social interaction test

In chronic PCP-treated mice, social interaction between adult mice was evaluated as described [33], with slight modifications. Mice were individually habituated to the observation apparatus (35 cm length × 25 cm width × 25 cm height) for 10 min for 2 consecutive days. Next, two unfamiliar test mice of the same genotype and treatment were placed in the apparatus, and social interaction behavior videotaped for 5 min. Time spent in active social interaction such as sniffing and following the partner, mounting, and crawling under/over the partner was measured by trained blinded observers.

### Novel object recognition memory test

Novel object recognition memory was evaluated as described [33,34], with slight modifications [47]. Mice were individually habituated to the observation box (30 cm length × 20 cm width × 20 cm height) for 10 min for 3 consecutive days. Next, a training session was performed, and mice were allowed to explore the observation box containing two different objects for 10 min. After 24 h, the retention test session was conducted, and each mouse was placed back in the observation box with a familiar object (presented in the training session) and a novel object. Behavior of the mice was videotaped and evaluated by trained blinded observers. Preference indices were calculated as the ratio of time spent exploring the novel object vs. the total time spent exploring both familiar and novel objects, and used as a dependent measure of recognition memory.

### Microarray analysis

Prefrontal cortex, hippocampus, and striatum were manually dissected from the brains of nine each Dys1A-Tg and wild-type mice. Three samples were pooled and subjected to GeneChip mouse genome 430 2.0 arrays (Affymetrix, Tokyo, Japan) which is one of the most comprehensive whole mouse genome expression array. A total of 18 hybridization experiments were performed according to the manufacturer’s instructions, and data analyzed using GeneChip Operating Software (GCOS) v1.1.1. GCOS was used to calculate the signal intensity and percent present calls on hybridized chips. Fold change of individual genes between Dys1A-Tg and wild-type mice are presented as the ratio of normalized gene expression values in Dys1A-Tg vs. wild-type mice.

### Statistical analyses

Statistical analysis was performed using StatView (SAS Institute Japan Ltd., Tokyo, Japan). Significant differences were determined by the Student’s *t*-test, Mann–Whitney *U* test, *χ*^2^ test or two- or three-way, factorial or repeated-measures ANOVA with genotype, drug, and time as factors of variation. Tukey–Kramer post-hoc tests were also performed after significant main effects for genotype, drug, or interaction between genotype × drug were observed. The threshold for statistical significance was defined as *P* < 0.05.
